# Imaging MS in Toxicology: An Investigation of Juvenile Rat Nephrotoxicity Associated with Dabrafenib Administration

**DOI:** 10.1007/s13361-015-1103-4

**Published:** 2015-03-25

**Authors:** M. Reid Groseclose, Susan B. Laffan, Kendall S. Frazier, Angela Hughes-Earle, Stephen Castellino

**Affiliations:** 1 Drug Metabolism and Pharmacokinetics, GlaxoSmithKline, Research Triangle Park, NC 27709 USA; 2 Safety Assessment, GlaxoSmithKline, King of Prussia, PA 19406 USA

**Keywords:** Imaging, MS, MALDI, LDI, Nephrotoxicity, Dabrafenib kidney deposits

## Abstract

As part of an investigative nephrotoxicity study, kidney tissues from juvenile rats orally administered dabrafenib at different age intervals between postnatal day (PND) 7 to 35 were investigated by MALDI and LDI imaging mass spectrometry (IMS) to determine the chemical composition of tubular deposits. In the youngest age group (PND 7-13), MALDI IMS demonstrated that a dabrafenib carboxylic acid metabolite was diffusely localized to the regions of tubular deposits (medulla and corticomedullary junction); however, no dabrafenib-related material was detected directly from the deposits. Rather, the LDI IMS analysis determined that the deposits were composed primarily of calcium phosphate. Based on these data, the dabrafenib associated nephrotoxicity, including the formation of tubular deposits, was determined to be age dependent. Furthermore, immature renal function was hypothesized to be responsible for the susceptibility of the youngest pups.

**Electronic supplementary material:**

The online version of this article (doi:10.1007/s13361-015-1103-4) contains supplementary material, which is available to authorized users.

**Graphical Abstract:**
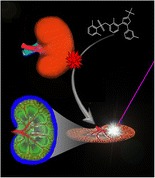
ᅟ

## Introduction

A mechanistic understanding of nonclinical toxicological events in drug development can be important in determining the human relevance and driving an effective risk assessment strategy. In such cases, the ability to investigate the molecular and cellular processes at the basis of the toxicological and pathologic findings induced by candidate drugs is critical in gaining the required mechanistic insight [1]. There are many analytical challenges associated with toxicological investigations aimed at examining the relationship between pathogenesis and the tissue distribution of a drug and its metabolites. In this application, LC-MS analysis of tissue homogenate extracts can be of limited value because the spatial information of analytes is lost. In some instances, tissue homogenate concentrations can be misleading. For example, if an analyte within the tissue is highly localized, the homogenization process will serve as a dilution and one may ignore a possible pathologic association because the measured tissue homogenate concentration is relatively low. Conversely, if an analyte is determined to have a high tissue homogenate concentration, one may be incorrectly drawn to make an association with tissue pathology.

Matrix-assisted laser desorption/ionization (MALDI) imaging mass spectrometry (IMS) has emerged as a powerful analytical tool for conducting mechanistic investigations into the tissue distribution of drugs and their metabolites [2–4]. In MALDI IMS, mass spectra are acquired across the surface of a tissue section creating a dataset from which a two-dimensional ion intensity image of any of the detected molecular species can be generated. The power of IMS lies in the ability to directly overlay the molecular information from the mass spectrometric analysis with the underlying tissue section to allow correlative visual comparisons of molecular and histologic information. The successful utilization of IMS in investigative toxicology studies is dependent on a carefully crafted and executed study design.

Dabrafenib (DAB), synonymous with TAFINLAR and GSK2118436, is an ATP-competitive inhibitor of RAF kinase activity. RAF kinases are a family of serine/threonine specific protein kinases involved in the mitogen-activated protein kinase (MAPK) pathway, which is a signaling cascade that mediates cell proliferation, differentiation, and survival [5]. Genetic mutations in various components of the MAPK pathway have been implicated in the progression of many human cancers, including melanoma, where *BRAF* mutation is present in around 50% of malignant tumors [6]. Dabrafenib has been approved in adults for the treatment of *BRAF* V600E mutation positive tumors as monotherapy and combination therapy with trametinib, an allosteric noncompetitive inhibitor of mitogen-activated protein kinases 1 and 2 (MEK1/MEK2).

In support of dabrafenib pediatric development, several clinical and nonclinical studies were conducted, including toxicity studies in the juvenile rat. In the definitive juvenile rat toxicity study, DAB-induced effects on the developing kidney were observed (Laffan et al., manuscript in preparation). No adverse kidney effects have been observed in adult animal studies with dabrafenib. Renal microscopic findings in the juvenile rats included tubular deposits, increased incidence of cortical cysts, tubular dilation, and increased incidence of tubular basophilia. Renal toxicity occurred with greater incidence and severity in younger pups, when dosing was initiated during the preweaning stage [before postnatal day (PND) 21], compared with older pups, where dosing was initiated postweaning (PND 22 or later). The tubular deposits were considered to be responsible for the pathogenesis of the additional renal findings in the proximal segments of the kidney due to the effects of obstructive nephropathy. The formation of renal deposits in the juvenile kidney was not anticipated and the mechanism was not well understood; the potential for these tubular deposits to be derived directly from DAB and/or its metabolites could not be ruled out.

The molecular composition of renal calculi can be indicative of the pathogenesis leading to their formation [7]. Xenobiotics have been shown to be responsible for the formation of kidney deposits, either directly through precipitation or by disruptions to kidney homeostasis (e.g., pH changes) that lead to the precipitation of endogenous components [8, 9]. Furthermore, the differentiation of nephrotoxicity by developmental stage suggests that the ontogeny associated with xenobiotic elimination routes may play a role in the formation of tubular deposits.

In this report, we present results from an additional study conducted to further examine the potential age sensitivity to dabrafenib induced renal toxicity initially observed in the definitive juvenile rat study. This investigative study combined multiple analytical approaches, including histopathologic assessment, specialized clinical chemistry, and urinalysis, in addition to mass spectrometric-based analyses. Here, we highlight the results of the IMS experiments used to analyze the kidney tissue distribution of DAB and its metabolites and determine the molecular composition of the renal deposits.

## Experimental

### Materials and Reagents

All reagents were purchased from Sigma-Aldrich (St. Louis, MO, USA) unless otherwise stated. 2,5-Dihydroxybenzoic acid (DHB, purity 98%) was purchased from Sigma Aldrich and purified by recrystallization prior to use. Calcium phosphate, dibasic (purity >98.5%) was purchased from Sigma-Aldrich and calcium oxalate (purity > 99.999%) was purchased from Alfa Aesar (Ward Hill, MA, USA). Indium tin oxide (ITO) coated glass microscope slides was purchased from Bruker (Billerica, MA, USA). Dabrafenib (DAB, GSK2118436A; N-{3-[5-(2-amino-4-pyrimidinyl)-2-(1,1-dimethylethyl)-1,3-thiazol-4-yl]-2-fluorophenyl}-2,6-difluorobenzene sulfonamide) from batch number JW211647-166P, 99.4% purity (% area) was supplied by GlaxoSmithKline and was stable when stored refrigerated (5°C) and protected from light for the duration of the study. Suspensions at concentrations of 2 and 10 mg/mL in 0.5% hydroxypropyl-methylcellulose (K15M) with 0.1% Tween 80 were dispensed weekly and were stable when stored refrigerated (5°C) for at least 15 d and protected from light.

### Study Design

All animal procedures were conducted in an American Association for the Accreditation of Laboratory Animal Care (AALAC)-accredited facility at GlaxoSmithKline (GSK) in accordance with GSK policies on the care, welfare, and treatment of laboratory animals, and they were reviewed and approved by GSK’s Institutional Animal Care and Use Committee (IACUC) as appropriate. Twenty-four time-mated female Crl:CD-1 (ICR)BR rats were obtained for this study from Charles River Laboratories Inc., (Raleigh, NC, USA). Mated females or parturient females and their litters were housed individually in plastic boxes containing Alpha-dri bedding (Shepherd Specialty Papers, Inc., Kalamazoo, MI, USA), except during the period of urine collection, when they were housed individually in stainless steel cages. Throughout the study, all animals were housed in a controlled environment (64°F to 79°F; 30% to 70% relative humidity) with an approximate 12-hour light/12-hour dark cycle. Litters were culled to 10 males/sex/litter on PND 4, weaned on PND 21, and then group housed for the remainder of the study. Since the renal findings occurred at a similar incidence in the males and females in the definitive study, only males were evaluated in this study. To accommodate the two dose levels with five different treatment intervals and to control for maternal care, five litters each (10/males per litter) were assigned to either the vehicle or the test-article group; then, two pups each from five litters were arbitrarily assigned different treatment periods to comprise the 10 groups.

Treatment periods were from PND 7 to 13, PND 14 to 21, PND 22 to 27, PND 28 to 35, or PND 7 to 35 (Table 1). Each group consisted of N = 10 juvenile male rats administered either vehicle control (Groups 1–5) or 10/20 mg/kg/d DAB (Groups 6–10) by oral gavage. The dose level was 10 mg/kg/day from PND 7 to PND 21, and then increased to 20 mg/kg/d from PND 22 to PND 35 to recapitulate the definitive juvenile toxicity study (Laffan et al., manuscript in preparation) in which the dose was increased as the pups aged to maintain consistent systemic exposure. For simplicity, individual juvenile rats are identified throughout the text by their group number (G1–10) and a unique rat number (R1–10) (e.g., G6R1). The PND x–x format is used as shorthand for the various DAB dosing time frames.

**Table 1:** Summary of Study Design and Dosing Regimen

Group number	Treatment	Treatment period (PND)	Day of necropsy (PND)	Dose level (mg/kg/d)
1	Vehicle	PND 7–13	PND 14	0
2	Vehicle	PND 14–21	PND 22	0
3	Vehicle	PND 22–27	PND 28	0
4	Vehicle	PND 28–35	PND 36	0
5	Vehicle	PND 7–35	PND 36	0
6	DAB	PND 7–13	PND 14	10
7	DAB	PND 14–21	PND 22	10
8	DAB	PND 22–27	PND 28	20
9	DAB	PND 28–35	PND 36	20
10	DAB	PND 7–35	PND 36	10/20

Termination occurred 1 d after the treatment period ended for each dose group (i.e., approximately 24 h after the last dose). At necropsy, the right kidney was fixed by immersion in 10% neutral buffered formalin and processed for light microscopic examination. The left kidney was bisected transversely at the hilus, placed in a plastic cryogenic mold (both cut surfaces side down), wrapped in foil and snap frozen in liquid nitrogen, and stored in a freezer set to maintain –70°C until all study samples were collected and shipped on dry ice for MALDI IMS analysis. Upon receipt, prior to sectioning, all tissues were maintained at –80°C.

### Tissue Sectioning

Thin sections (6 μm) from the midline of the kidney tissues were collected in a cryostat (–20°C) and mounted onto ITO coated glass microscope slides. Sections serial to those collected for IMS were taken for hematoxylin and eosin (H&E) staining to enable correlated histologic analysis. Prior to matrix application, all tissue sections collected for IMS and all H&E stained sections were scanned at high magnification (20x – 40x) using an Aperio Scanscope CS (Leica, Buffalo Grove, IL, USA).

### Matrix Application

For the MALDI IMS experiments, DHB was applied using either a custom built sublimation apparatus or TM Sprayer (HTX Technologies, Carrboro, NC, USA). For the MALDI IMS experiments investigating the distribution of DAB and its metabolites (data shown in Figures 2 and Figure 3), DHB (50 mg/mL) in water/methanol (1:1 v/v) was applied using a TM Sprayer (HTX Technologies) with the following parameter settings: number of passes = 8; flow rate = 0.1 mL/min; spray temperature = 70°C; velocity = 1333 mm/min; track spacing = 3.0 mm; track offset = 1.5 mm. For sublimation (data shown in Figure 4), the system was operated under vacuum (~300 mTorr) and a heating mantle was used to heat the chamber containing DHB to ~130°C for approximately 15 min. Based on weight, approximately 12 mg of DHB was applied to the slides. Following sublimation, slides were incubated in a chamber saturated with methanol for approximately 20 min. No additional preparation or processing was conducted on the tissue sections analyzed by laser desorption ionization (LDI).

### Imaging Mass Spectrometry (IMS)

Two separate methods of ionization were used to acquire the IMS datasets: laser desorption ionization (LDI) and matrix-assisted laser desorption/ionization (MALDI).

All MALDI and LDI IMS experiments were conducted using a Bruker Solarix 7T Fourier transform-ion cyclotron resonance mass spectrometer (FT-ICR MS). Images were acquired at spatial resolutions ranging from 10 to 100 μm. Mass spectra were acquired both in full scan mode (*m/z* 80–1000) and continuous accumulation of selected ions (CASI) mode where a narrow mass range (*m/z* 460–620) was selected to enhance sensitivity for DAB-related material. LDI IMS experiments were conducted by increasing the laser power setting ~2 × –3× higher than the laser power settings typically used for MALDI MS acquisition of DHB. All ion images were generated using FlexImaging v4.0 software from the raw data. Unless specified otherwise, all ion images are displayed with a mass tolerance of +/– 0.0005 Da, minimum intensity threshold of 2%, and a maximum intensity threshold of 70%. Ion images exported from FlexImaging were processed to remove the black background and overlayed with the corresponding H&E images using Photoshop CC (Adobe, San Jose, CA, USA).

When analyzed by MALDI MS, DAB, carboxy-dabrafenib (CDAB), hydroxy-dabrafinib (HDAB), and desmethyl-dabrafenib (DDAB) exhibit peaks corresponding to a double bond reduction or ring opening hydrogenation [M + H + 2H]^+^ in addition to the [M + H]^+^ peak (Electronic Supplementary Material, Figure S2).

Additionally, when analyzed by MALDI MS, extensive conversion (up to 90%) of CDAB to DDAB occurs through decarboxylation (Electronic Supplementary Material, Figure S3). The LC-MS quantification showed that CDAB was the predominant species in the kidney of the PND 7–14 rats and the concentration of native DDAB was negligible. Given that the conversion of CDAB to DDAB was shown to be consistent across a large range of concentrations the signal for DDAB is used as a surrogate for CDAB and referred to CDAB* throughout the text.

## Results and Discussion

### Light Microscopic Histopathologic Evaluation

Increased kidney weights and microscopic renal findings were of highest incidence and severity in pups treated during preweaning [Group 6 (PND 7 to 13) and Group 7 (PND 14 to 21)] compared with pups that initiated treatment postweaning [Group 8 (PND 22 to 27) and Group 9 (PND 28 to 35)]. Pups treated continuously from PND 7 to 35 (Group 10) presented most of the effects noted during the preweaning period but findings, in total, were generally less severe. Notably, the tubular deposits were present at high incidence in pups treated anytime during preweaning in all three groups; PND 7 to 13, 14, to 21 and 7 to 35. Importantly, no tubular deposits were found in the groups dosed postweaning only (PND 22 to 27 and PND 28 to 35). A similar incidence of deposits were noted in pups treated continuously from PND 7 to 35, suggesting those deposits present prior to PND 22 persisted throughout the dosing period but did not increase with extended duration of dosing beyond weaning.

**Figure 1:**
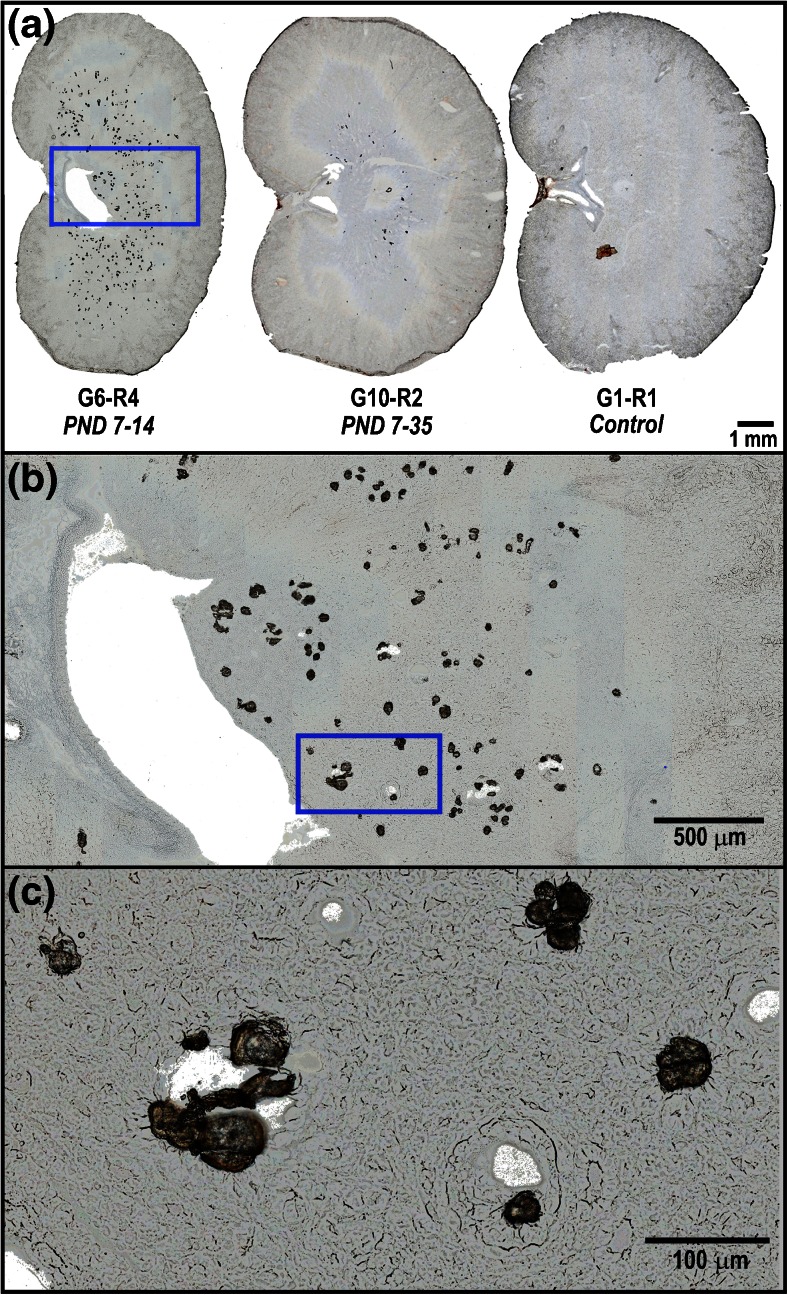
(**a**) Optical scan (unstained) of kidney tissue sections from juvenile rats: G6-R4 (DAB 10 mg/kg/d; PND 7–14), G10-R2 (DAB 10/20 mg/kg/d; PND 7–35), and G1-R1 (vehicle/PND 7–14), (**b**) 5× and (**c**) 20× magnification views from juvenile rat G6-R4 kidney section showing tubular deposits

The tubular deposits, primarily located in the medulla, were characterized by clear to deeply basophilic crystalline to mineralized material filling the lumina. Optical images (unstained) of representative kidney tissue sections from three juvenile rats are presented in Figure 1a showing the high density of tubular deposits observed in a PND 7–14 animal G6-R4 (left) in comparison to a PND 7–35 animal G10-R2 (middle), where markedly fewer deposits are still visible. No deposits were observed in the kidney tissues from the control animal G1-R1 (right). The deposits ranged in size from 10 to 100 μm, as shown in Figure 1b and c, which display magnified views of a selected region of the kidney (G6-R4).

Other DAB-related microscopic renal findings included minimal to moderate dilation of cortical and medullary tubules, increased incidence of cortical cysts, minimal to mild degeneration (especially cytoplasmic basophilia) of cortical and medullary tubules. These tubular deposits also resulted in the pathogenesis of secondary changes in more proximal segments of kidneys, leading to obstructive nephropathy.

### Renal Distribution of DAB and Metabolites

**Scheme 1:**
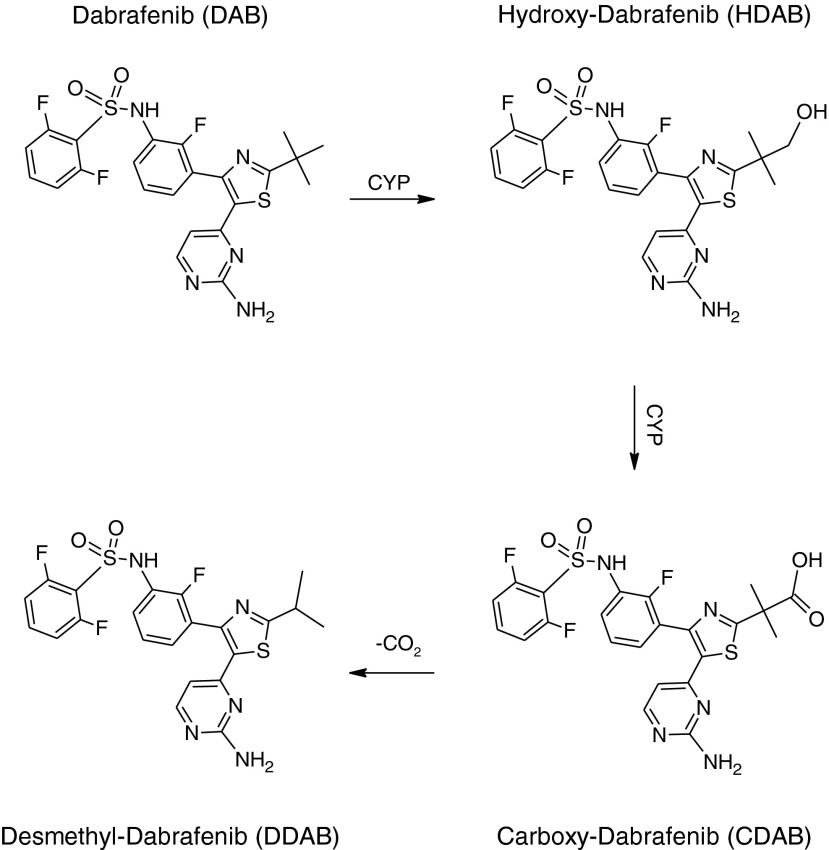
The primary metabolic pathway of DAB in humans and preclinical species

The primary metabolic pathway of DAB in humans and preclinical species involves an oxidation cascade starting with the *tert*-buty group (Scheme 1) [10]. Initial cytochrome P450 mediated oxidation results in the formation of HDAB followed by further oxidation to form CDAB. A non-enzymatic decarboxylation mechanism has been proposed for the formation of DDAB.

An LC-MS kidney tissue homogenate survey to characterize DAB and its metabolites was conducted on a representative subset of animals from each dose group (Electronic Supplementary Material, Figure S4). These data showed that the highest concentration of DAB-related material was present in the kidney tissues from the youngest rats [Group 6 ( PND 7–13)] with the predominant DAB-related species detected in this group (n = 4) being CDAB (mean: 8190 [6114–11,657] ng/g) followed by HDAB (mean: 442 [352–596] ng/g) and DDAB (mean: 72 [38–110 ng/g). DAB was not detected in the PND 7–13 samples by LC-MS. In the kidney tissues from the additional dosing groups, which were all assessed on PND 22 or older, DAB was not detected and markedly lower levels of DAB-related metabolites were present relative to the PND 7–13 group. The LC-MS data provides a temporal link between the quantity of DAB-related material (mainly CDAB) and the observed kidney injury in younger juvenile rats. However, these data provide no insight into the relationship between DAB or its metabolites and the tubular deposits, which were believed to be the primary source of injury.

**Figure 2:**
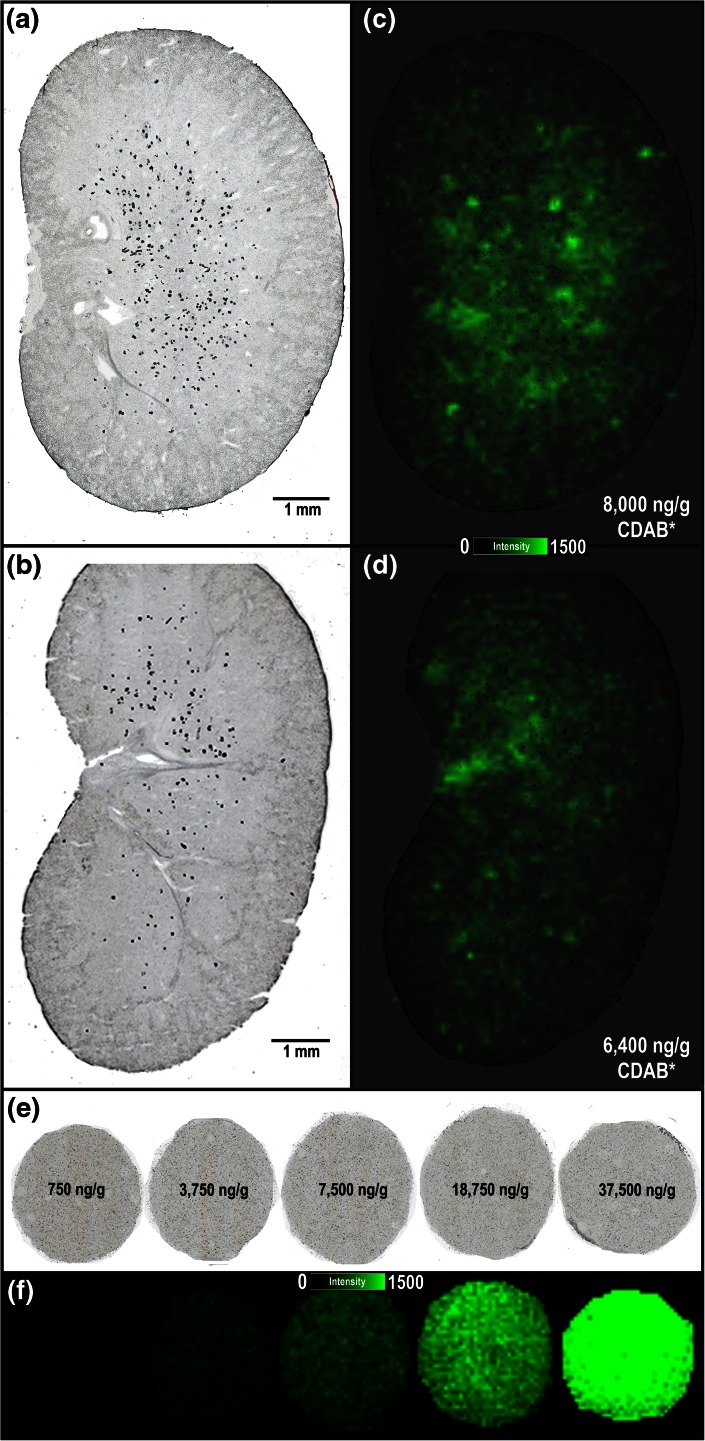
Optical scans of kidney tissue sections from PND 7–13 juvenile rats (**a**) G6-R2 and (**b**) G6-R3 analyzed by MALDI IMS in CASI mode (*m/z* 460–620) at 100 μm spatial resolution. (**c**) and (**d**) Respective ion images for CDAB* [M + 2H + H]^+^ (*m/z* 508.1083) and quantity (ng/g of tissue) predicted using tissue mimetic model. (**e**) Pre-analysis optical scan of tissue mimetic model cores with spiked concentration of CDAB labeled for each (**f**) ion image for CDAB* [M + 2H + H]^+^ (*m/z* 508.1083) from tissue model analyzed under same conditions as the kidney tissue sections

Our initial MALDI IMS experiments were focused on determining the distribution of DAB and its metabolites in the kidney given the literature precedence for drug or drug metabolite precipitation as the cause of tubular deposits [8, 9, 11]. Figure 2 displays the distribution of CDAB* in kidney tissue sections from two PND 7–13 juvenile rats (G6-R2 and G6-R3). In both animals, CDAB* was diffusely localized predominantly to the inner medulla and pelvic regions with less intense signals from the outer medulla and cortex (Figure 2c and d). An estimate of the tissue concentration of CDAB* in these section was generated using a tissue mimetic model (Figure 2e and f). As previously described [12], the tissue mimetic model can be used to estimate drug quantities by comparing the average ion signal detected by MALDI IMS for an analyte in a tissue section to a calibration curve analyzed in parallel. For G6-R2, the concentration of CDAB was estimated to be 8000 ng/g by the MALDI IMS tissue model approach compared with 11,600 ng/g by LC-MS and for G6-R3, 6400 ng/g by MALDI IMS compared with 6100 ng/g by LC-MS. Considering that the MALDI IMS results are generated on a single tissue section (<1 mg) compared with the LC-MS results, which are generated on extracts from half of a kidney (>200 mg), these results are in relatively good agreement, and differences in the estimated quantities might be, at least partially, attributed to regional concentration differences in the kidney.

**Figure 3:**
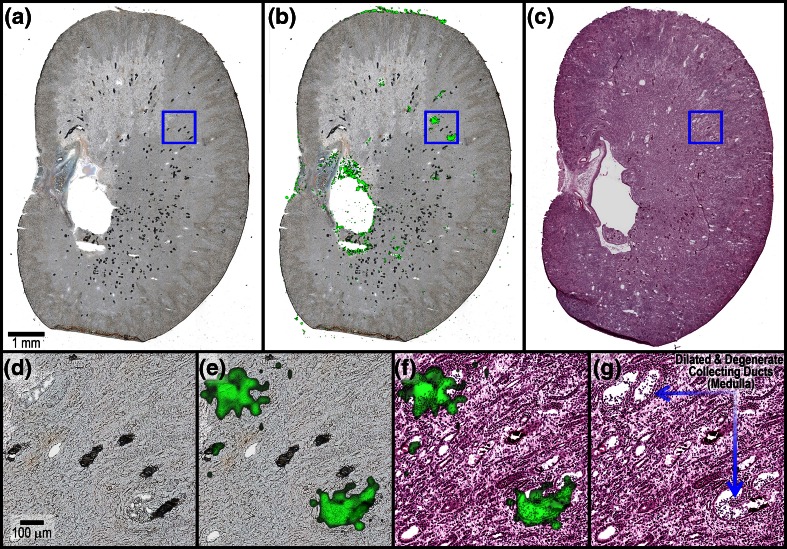
(**a**) Optical scan of kidney tissue sections from PND 7–13 juvenile rat G6-R2 analyzed by MALDI IMS in CASI mode (*m/z* 460–620) at 25 μm spatial resolution. (**b**) Ion image for CDAB* [M + 2H + H]^+^ (*m/z* 508.1083). (**c**) Serial H&E. (**d**) 10× magnification of outlined region in optical scan. (**e**) Magnified view of CDAB* ion image co-registered with optical scan. (**f**) Magnified view of CDAB* ion image co-registered with H&E. (**g**) Histopathology annotated H&E

The low spatial resolution (100 μm) imaging experiments displayed in Figure 2 helped correlate the distribution of DAB-related material to the gross anatomical features of the kidney sections with high sensitivity. In addition to these, several high spatial resolution imaging experiments were conducted to provide a more detailed view of the relationship between the CDAB* and the tissue histology and rule out a direct association with the tubular deposits. For example, Figure 3 displays the distribution of CDAB* at 25 μm spatial resolution in a kidney tissue section from a PND 7–13 juvenile rat (G6-R2). The focally intense regions throughout the medulla where CDAB* is detected with highest intensity (Figure 3b) correspond to dilated and degenerate collecting ducts in the serial H&E (Figure 3c). This is more clearly illustrated in the magnified views (Figure 3d–g) showing the overlays of the ion image for CDAB* with the optical scan and H&E. Here, the distribution of CDAB* is shown to be highly localized to the lumen of the damaged collecting ducts. Importantly, neither CDAB* nor any other DAB-related material was detected directly from the tubular deposits in any of the imaging experiments conducted.

Low intensity levels of HDAB were detected in the cortical regions of the PND14 assessed kidney tissues (data not shown); however, neither DAB nor any additional DAB-metabolites were observed. No DAB-related material was detected by MALDI IMS or LC-MS in kidney tissues from the PND 7–35 group despite the presence of tubular deposits.

### Renal Deposit Characterization

**Figure 4:**
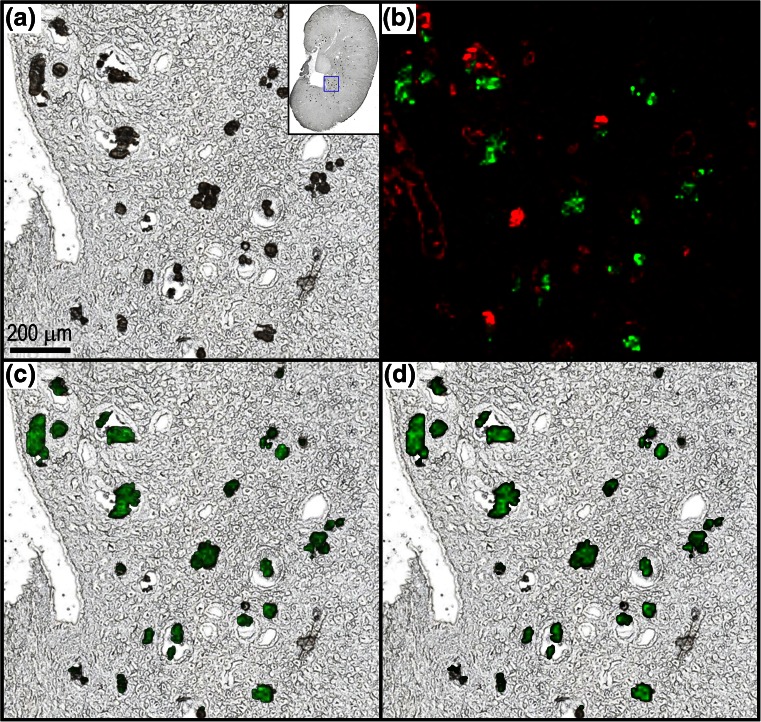
(**a**) Optical scan of kidney tissue sections from PND 7–13 juvenile rat G6-R4 analyzed by MALDI IMS in fullscan mode (*m/z* 100–1000) at 10 μm spatial resolution. (**b**) Ion image for [3DHB – H + Ca]^+^ (*m/z* 501.0339) in green, and glycerophosphocholine [M + K]^+^ (*m/z* 296.0659) in red. (**c**) Ion image for [2DHB – 2H + Ca]^•+^ (*m/z* 345.9999). (**d**) Ion image for [2DHB – O – 2H + Ca]^•+^ (*m/z* 330.0047)

Determining the composition of the deposits was important for insight into the mechanism of DAB-related nephrotoxicity in the preweaning rats. The MALDI IMS experiments revealed numerous peaks detected exclusively from the tubular deposits. Based on accurate mass and isotopic distribution, the deposit-specific peaks were identified as various DHB-calcium clusters. For example, Figure 4 displays ion images for several of these high intensity matrix-calcium clusters that were detected exclusively from the deposits in a section from PND 7–13 animal G6-R2. A similar pattern of cluster formation was reported by Dubois et al.[13], where it was shown that DHB readily forms clusters in the presence of high concentrations of calcium. This was useful information, although not surprising, as calcium salts, including calcium oxalate and calicum phosphate, are known to be present in approximately 80% of renal calculi (i.e., calcium based deposits) [14]. However, distinguishing between calcium oxalate and calcium phosphate deposits can be important because their formation is driven by different pathogenic mechanisms [14–16].

**Figure 5:**
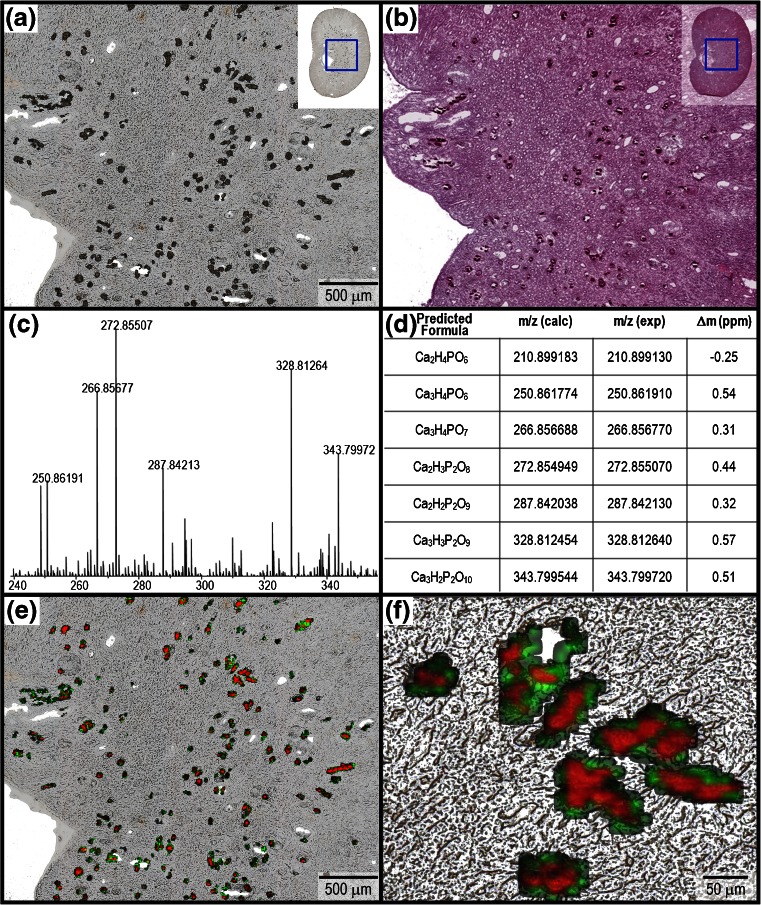
(**a**) Optical scan of a region of a kidney tissue section from PND 7–13 juvenile rat G6-R2 analyzed by LDI IMS without matrix at 10 μm spatial resolution. (**b**) Serial H&E. (**c**) Partial view of the spectrum generated by averaging all spectra from pixels localized to the deposits. (**d**) Peak identification table for the most abundant peaks (labeled) in the mass spectrum. (**e**) Overlayed ion images for *m/z* 272.8550 (Ca_2_H_3_P_2_O_8_) in red, and *m/z* 228.9097 (Ca_2_H_6_PO_7_) green. (**f**) Magnified view (20×)

Further characterization of the deposits was conducted using LDI IMS, where regions of the kidney tissues were analyzed in the absence of a MALDI matrix. From these experiments, it was possible to detect a wide variety of calcium phosphate species directly from the tubular deposits. For example, targeted regions of a kidney tissue section from a PND 7–13 juvenile rat G6-R2 were analyzed by LDI IMS and are shown in Figure 5a and b. The spectra generated from the deposits contained a large number of peaks (Figure 5c), which, based on accurate mass and isotope pattern, were primarily identified as various hydrated calcium phosphate species (Figure 5d). Figure 5e and f display the ion images for two of these calcium phosphate species: *m/z* 272.8549 (Ca_2_H_3_P_2_O_8_) in red and *m/z* 228.91757 (Ca_2_H_6_PO_7_) in green. Interestingly, these two species are detected with different distributions from within the deposits, where *m/z* 228.91757 is localized to the perimeter and *m/z* 272.8549 to the inner area. It is known that the composition of renal calculi can consist of heterogeneous layers attributable to the processes of nucleation, crystal growth, and aggregation [17].

**Figure 6:**
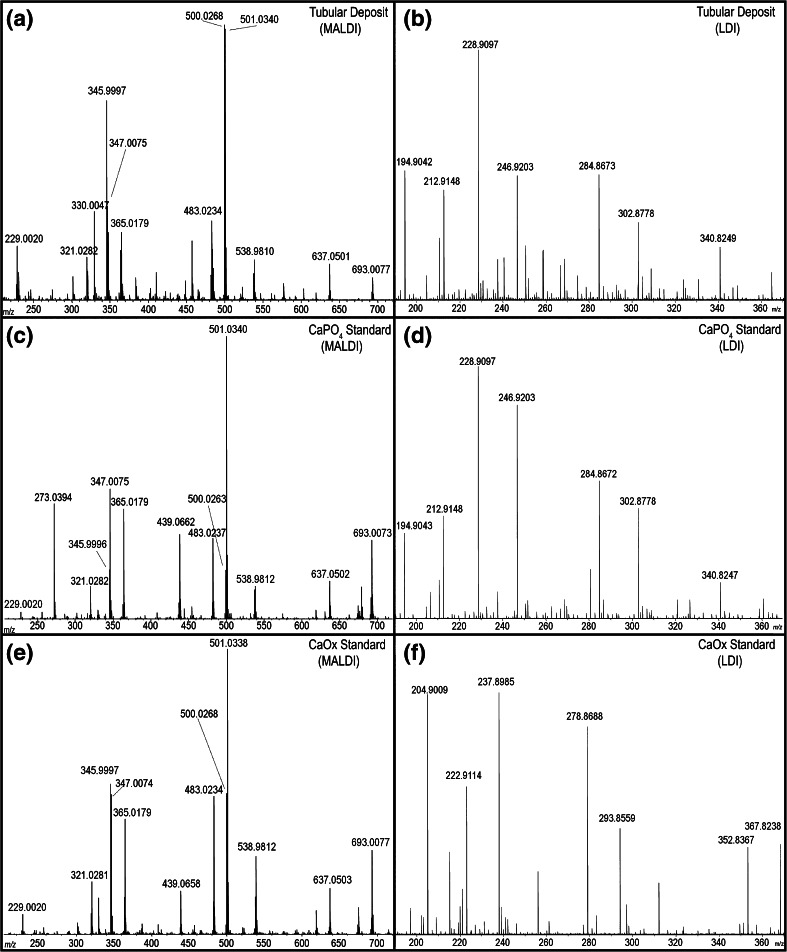
Saturated soluions of calcium phosphate and calcium oxalate in water were precipitated onto an ITO coated glass slide by pipetting 1 μL (×3). For MALDI MS, DHB matrix was applied to the kidney tissue section using a TM Sprayer and to the standards by pipetting 1 μL [50 mg/mL H_2_O/MeOH (1:1 v/v)]. No matrix was applied for the LDI mass spectra. Both MALDI and LDI fullscan (*m/z* 100–1000) mass spectra were manually acquired in positive ion mode. MALDI mass spectra generated by averaging 50 scans (50 laser shots each) from (**a**) tubular deposit in a kidney tissue section from PND 7–13 juvenile rat G6-R2. (**c**) Calcium phosphate standard and (**e**) calcium oxalate standard. LDI mass spectra generated by averaging 100 scans (500 laser shots each) from (**b**) tubular deposit in a juvenile kidney tissue section (G6-R2), (**d**) calcium phosphate, and (**f**) calcium oxalate

In order to verify the proposed peak assignments from the tubular deposits, tissue section profiles were compared with standards of calcium oxalate and calcium phosphate by MALDI and LDI MS (Figure 6). A peak list table is shown in Table 2 as a reference for the highest intensity ions detected in each spectrum. Figure 6a, c, and e display representative MALDI mass spectra generated from the kidney deposits (G6-R2), the calcium phosphate standard, and the calcium oxalate standard, respectively. The MALDI mass spectra generated for these three samples are very similar, where the major species detected are DHB-calcium adducts. In contrast, the LDI mass spectra (Figure 6b, e, and f) provide a clear distinction, where the spectra from the renal deposits and calcium phosphate standard show a similar distribution of calcium phosphate species. Calcium hydroxide clusters are the predominant species in LDI mass spectrum generated from the calcium oxalate standard.

**Table 2:** Reference Table for Highest Intensity Peaks Detected in Spectra Displayed in Figure 6

MALDI MS			*Tubular Deposit*			*CaPO*_*4*_ *Standard*			*CaOx Standard*		
Predicted Formula	Putative Molecular Species	*m/z* (calc)	*m/z* (exp)	Rel. Int. (%)	Δm (ppm)	*m/z* (exp)	Rel. Int. (%)	Δm (ppm)	*m/z* (exp)	Rel. Int. (%)	Δm (ppm)
C_7_H_9_CaO_6_	[DHB – H + Ca]^+^∙2H_2_O	229.00196	229.00199	21	–0.15	229.00196	3	–0.02	229.00200	7	–0.20
C_14_H_10_CaO_8_	[2DHB – 2H2O – 2H + Ca]^•+^∙2H_2_O	345.99961	345.99955	71	0.17	345.99963	18	–0.06	345.99954	52	0.20
C_14_H_11_CaO_8_	[2DHB – 2H_2_O – H + Ca]^+^∙2H_2_O	347.00744	347.00739	33	0.13	347.00741	46	0.07	347.00737	49	0.19
C_21_H_16_CaO_12_	[3DHB – 2H_2_O – 2H + Ca]^•+^∙2H_2_O	500.02622	500.02605	100	0.34	500.02643	17	–0.42	500.02624	49	–0.04
C_21_H_17_CaO_12_	[3DHB – 2H_2_O – H + Ca]^+^∙2H_2_O	501.03404	501.03400	97	0.09	501.03389	100	0.31	501.03371	100	0.66
C_21_H_15_Ca_2_O_12_	[3DHB – 3H_2_O – 3H + 2Ca]^+^∙3H_2_O	538.98098	538.98096	18	0.04	538.98103	12	–0.09	538.98105	27	–0.12
C_28_H_21_CaO_15_	[4DHB – 3H_2_O – H + Ca]^+^∙2H_2_O	637.05009	637.05022	15	-0.21	637.05019	11	–0.16	637.05034	16	–0.40
C_28_H_21_Ca_2_O_16_	[4DHB – 3H_2_O – 3H + 2Ca]^+^∙3H_2_O	693.00759	693.00802	13	–0.62	693.00732	28	0.39	693.00796	29	–0.53
LDI MS			*Tubular Deposit*			*CaPO*_*4*_ *Standard*			*CaOx Standard*		
Predicted Formula	Putative Molecular Species	*m/z* (calc)	*m/z* (exp)	Rel. Int. (%)	Δm (ppm)	*m/z* (exp)	Rel. Int. (%)	Δm (ppm)	*m/z* (exp)	Rel. Int. (%)	Δm (ppm)
H_4_Ca_2_O_5_P	[Ca_2_PO_3_]^+^· 2H_2_O	194.90427	194.90424	47	0.16	194.90425	32	0.08	ND	–	–
H_6_Ca_2_O_6_P	[Ca_2_PO_3_]^+^· 3H_2_O	212.91483	212.91481	40	0.12	212.91482	37	0.07	ND	–	–
H_6_Ca_2_O_7_P	[Ca_2_PO_4_]^+^ · 3H_2_O	228.90975	228.90973	100	0.06	228.90973	100	0.09	ND	–	–
H_8_Ca_2_O_8_P	[Ca_2_PO_4_]^+^ · 4H_2_O	246.92031	246.92029	45	0.11	246.92029	77	0.10	ND	–	–
H_6_Ca_3_O_8_P	[Ca_3_PO_4_(OH)_2_]^+^ · 2H_2_O	284.86725	284.86729	46	–0.13	284.86723	32	0.08	ND	–	–
H_8_Ca_3_O_9_P	[Ca_3_PO_4_(OH)_2_]^+^ · 3H_2_O	302.87782	302.87777	28	0.15	302.87776	40	0.20	ND	–	–
H_6_Ca_4_O_9_P	[Ca_4_PO_4_(OH)_4_]^+^ · H_2_O	340.82476	340.82491	19	–0.44	340.82471	13	0.13	ND	–	–
H_5_Ca_3_O_5_	[Ca_3_(OH)_5_]^+^	204.90092	204.90091	9	0.05	204.90092	4	0.00	204.90090	100	0.11
H_7_Ca_3_O_6_	[Ca_3_(OH)_5_]^+^· H_2_O	222.91149	222.91146	4	0.11	222.91148	3	0.05	222.91150	55	–0.06
H_6_Ca_3_O_7_	[Ca_3_(OH)_6_ + O]^•+^	237.89858	237.89855	15	0.11	237.89856	10	0.07	237.89860	89	–0.10
H_7_Ca_4_O_7_	[Ca_4_(OH)_7_]^+^	278.86899	278.86898	5	0.05	278.86897	2	0.09	278.86900	76	–0.03
H_6_Ca_4_O_8_	[Ca_4_(OH)_6_ + 2O]^•+^	293.85608	293.85605	4	0.12	293.85600	2	0.27	293.85610	39	–0.06
H_9_Ca_5_O_9_	[Ca_5_(OH)_10_]^+^	352.83706	ND	–	–	ND	–	–	352.83710	32	–0.10
H_8_Ca_5_O_10_	[Ca_5_(OH)_8_ + 2O]^•+^	367.82415	ND	–	–	ND	–	–	367.82420	33	–0.13

These experiments led us to the conclusion that the tubular deposits were primarily composed of calcium phosphate and were devoid of DAB-related material. Additionally, the molecular composition of the deposits as detected by MALDI and LDI MS was consistent between the PND 7–14 and PND 7–35 treatment groups (data not shown). The differences in the LDI mass spectra from the imaging experiment (Figure 5c) and the profiling experiment (Figure 6a) are likely due to the higher laser energy (less attenuation) used for acquistion of the data in Figure 5 relative to the data in Figure 6. This was consistently observed in the LDI MS experiments, where differences in laser energy affected the intensity distribution of the various calcium phosphate clusters detected, but not the overall composition of the spectra. The putative molecular species listed in Table 2 were generated using a logical combination of the elements in the predicted ion formula; an exhaustive structural characterization of the species detected, beyond the LDI and MALDI MS, was not conducted.

### Mechanistic Hypothesis for Nephrotoxicity

Determining the precise etiology of DAB juvenile nephrotoxicity was beyond the scope of this study design; however, some mechanistic insights for risk assessment can be gained from these data. Based on the histopathologic assessment alone, juvenile rats administered DAB exhibit an age-dependent nephrotoxic response. Specifically, the incidence and severity of nephrotoxicity was greatest in rats where DAB administration was initiated in younger rat pups during the preweaning phase. For older pups that began receiving DAB postweaning, there were fewer and less severe kidney findings. These observations suggest a unique sensitivity of the developing kidney to the nephrotoxic potential of DAB in rats.

The LC-MS analysis showed markedly higher quantites of CDAB in the kidneys of the youngest rats (PND 7–13) relative to the older dosing groups. A similar pattern was observed in a previous juvenile rat toxicokinetic study with the same dosing regimen, where CDAB was the predominant circulating metabolite with 3 to 5 times higher dose-dependent plasma exposure during the preweaning period compared with postweaning (Laffan et al, manuscript in preparation).

Several factors may have contributed to the increased renal tissue concentration of DAB-related material in the youngest pups (PND 7–13). The process of bile formation in rats (and humans) is known to be immature at birth and therefore biliary elimination of many drugs is impaired during the neonatal period [18–21]. The biliary clearance system gradually develops over the first several postnatal weeks to adult levels. Given that fecal elimination (which includes biliary) is the major clearance route for DAB and its metabolites in adult rats [10], the increased systemic and kidney concentrations in the youngest pups may be reflective of undeveloped biliary excretion.

In the preweaning rats administered DAB, the primary site of toxicologic injury was the tubular epithelial cells in the distal nephron (collecting duct) and the formation of tubular deposits in the inner medulla. The formation of drug-induced kidney deposits can either be a direct result of drug or metabolite precipitation or the drug or metabolites may cause physiological perturbations (e.g., changes in pH) that lead to the precipitation of endogenous components [11]. The MALDI and LDI IMS analyses support that the tubular deposits consisted primarily of calcium phosphate and did not contain any DAB-related material. Because the distal nephron is recognized to be a primary site of calcium homeostasis [22], it is possible that damage to the tubular epithelium resulting from DAB administration in the preweaning animals (Groups 6 and 7) could trigger localized disruption of the acid-base homeostasis resulting in formation of calcium phosphate calculi. Furthermore, the preweaning rats may be more susceptible to such an insult because of immature renal function. Animal studies have demonstrated an immaturity and decreased activity of several transporters related to the maintenance of acid-base homeostasis in the neonatal kidney [23]. Karashima et al. [24] found that in rats, carbonic anhydrase II levels increased from 14% to 40% of adult levels from the first to third postnatal week. Renal phosphate transport in rats also reaches maturity during the third postnatal week [25].

The deposits observed in the PND 7–35 extended treatment group are likely formed during the preweaning period (PND 7–21) based on the observation that there was no increase in the incidence and severity of the calculi formed when comparing these animals to the PND 7–13 treatment group. Because calculi are very slowly resorbed, once formed, tubular injury will likely persist through or after PND 35, even though the direct nephrotoxic effects of the compound appear to diminish after PND 22.

Determining whether the initial tubular injury was directly or indirectly caused by DAB or its metabolites was beyond the scope of this investigation. Nonetheless, given the markedly high levels of CDAB detected in the kidneys of the PND 7–13 animals, the localization of CDAB and deposits within the kidney, and the absence of CDAB in the kidneys of unaffected older animals, a direct link does seem plausible.

## Conclusions

Nonclinical studies in drug development are conducted to assess safety in animal models and build a risk assessment for patients. Toxicologists traditionally screen for adverse findings by histologic examination of tissues with light or even electron microscopy, in addition to monitoring for urine or plasma biomarkers of pathology. Imaging MS provides a platform to more directly link the chemistry and biology involved in the processes associated with toxicologic effects; specifically, overlaying the tissue distribution of drugs and their metabolites with the location of changes in tissue morphology. The application of both MALDI and LDI IMS in this study serves as an example of how this platform can impact drug discovery and development. IMS analysis of juvenile rat kidney tissues was able to add a deeper mechanistic understanding to the nephrotoxicity than LC-MS homogenate data alone, by characterizing the tissue distribution of the DAB metabolites and determining the chemical composition of the tubular deposits. From these data, along with histologic findings, a more complete risk assessment for pediatric treatment with dabrafenib is possible.

## Electronic supplementary material

Below is the link to the electronic supplementary material.

ESM 1 (DOCX 1242 kb)

## References

[1] Ahuja V, Sharma S (2014). Drug safety testing paradigm, current progress and future challenges: an overview. J. Appl. Toxicol..

[2] Castellino S, Groseclose MR, Wagner D (2011). MALDI imaging mass spectrometry: bridging biology and chemistry in drug development. Bioanalysis.

[3] Greer T, Sturm R, Li L (2011). Mass spectrometry imaging for drugs and metabolites. J. Proteom..

[4] Prideaux B, Stoeckli M (2012). Mass spectrometry imaging for drug distribution studies. J. Proteom..

[5] Trinh VA, Davis JE, Anderson JE, Kim KB (2014). Dabrafenib therapy for advanced melanoma. Ann. Pharmacother..

[6] Hauschild A, Grob JJ, Demidov LV, Jouary T, Gutzmer R, Millward M, Rutkowski P, Blank CU, Miller WH, Kaempgen E, Martin-Algarra S, Karaszewska B, Mauch C, Chiarion-Sileni V, Martin AM, Swann S, Haney P, Mirakhur B, Guckert ME, Goodman V, Chapman PB (2012). Dabrafenib in BRAF-mutated metastatic melanoma: a multicentre, open-label, Phase 3 randomised controlled trial. Lancet.

[7] Frazier KS, Seely JC, Hard GC, Betton G, Burnett R, Nakatsuji S, Nishikawa A, Durchfeld-Meyer B, Bube A (2012). Proliferative and nonproliferative lesions of the rat and mouse urinary system. Toxicol. Pathol..

[8] John R, Herzenberg AM (2009). Renal toxicity of therapeutic drugs. J. Clin. Pathol..

[9] Yarlagadda SG, Perazella MA (2008). Drug-induced crystal nephropathy: an update. Expert Opin. Drug Saf..

[10] Bershas DA, Ouellet D, Mamaril-Fishman DB, Nebot N, Carson SW, Blackman SC, Morrison RA, Adams JL, Jurusik KE, Knecht DM, Gorycki PD, Richards-Peterson LE (2013). Metabolism and disposition of oral dabrafenib in cancer patients: proposed participation of aryl nitrogen in carbon–carbon bond cleavage via decarboxylation following enzymatic oxidation. Drug Metab. Dispos..

[11] Matlaga BR, Shah OD, Assimos DG (2003). Drug-induced urinary calculi. Rev. Urol..

[12] Groseclose MR, Castellino S (2013). A mimetic tissue model for the quantification of drug distributions by maldi imaging mass spectrometry. Anal. Chem..

[13] Dubois, F., Knochenmuss, R., Steenvoorden, R., Breuker, K., Zenobi, R.: On the mechanism and control of salt-induced resolution loss in matrix-assisted laser desorption/ionization. Eur. J. Mass Spectrom. **2**, 167–172 (1996)

[14] Coe FL, Evan A, Worcester E (2005). Kidney stone disease. J. Clin. Invest..

[15] Mulay SR, Evan A, Anders HJ (2014). Molecular mechanisms of crystal-related kidney inflammation and injury. Implications for cholesterol embolism, crystalline nephropathies, and kidney stone disease. Nephrol. Dial. Transplant..

[16] Sakhaee K (2009). Recent advances in the pathophysiology of nephrolithiasis. Kidney Int..

[17] Lee T, Lin YC (2011). Mimicking the initial development of calcium urolithiasis by screening calcium oxalate and calcium phosphate phases in various urinelike solutions, time points, and Ph values at 37C. Cryst. Growth Des..

[18] Tomer G, Ananthanarayanan M, Weymann A, Balasubramanian N, Suchy FJ (2003). Differential developmental regulation of rat liver canalicular membrane transporters BSEP and MRP2. Pediatr. Res..

[19] St-Pierre MV, Stallmach T, Freimoser Grundschober A, Dufour JF, Serrano MA, Marin JJ, Sugiyama Y, Meier PJ (2004). Temporal expression profiles of organic anion transport proteins in placenta and fetal liver of the rat. Am. J. Physiol. Regul. Integr. Comp. Physiol..

[20] Fattah S, Augustijns P, Annaert P (2015). Age-dependent activity of the uptake transporters NTCP and OATP1b2 in male rat hepatocytes: from birth till adulthood. Drug Metab. Dispos..

[21] Gao B, St Pierre MV, Stieger B, Meier PJ (2004). Differential expression of bile salt and organic anion transporters in developing rat liver. J. Hepatol..

[22] Agus ZS, Chiu PJ, Goldberg M (1977). Regulation of urinary calcium excretion in the rat. Am. J. Physiol..

[23] Ingulli EG, Mak RH (2014). Growth in children with chronic kidney disease: role of nutrition, growth hormone, dialysis, and steroids. Curr. Opin. Pediatr..

[24] Karashima S, Hattori S, Ushijima T, Furuse A, Nakazato H, Matsuda I (1998). Developmental changes in carbonic anhydrase II in the rat kidney. Pediatr. Nephrol..

[25] Spitzer A, Barac-Nieto M (2001). Ontogeny of renal phosphate transport and the process of growth. Pediatr. Nephrol..

