# Supplementary material for: Imaging MS in Toxicology: An Investigation of Juvenile Rat Nephrotoxicity Associated with Dabrafenib Administration
Source: J Am Soc Mass Spectrom. 2015 Mar 25;26(6):887–98. doi: 10.1007/s13361-015-1103-4 (PMC4422858; doi:10.1007/s13361-015-1103-4)
Supplement: Supplementary file 1 — (DOCX 1242 kb) [file 13361_2015_1103_MOESM1_ESM.docx]

Imaging MS in Toxicology: An Investigation of Juvenile Rat Nephrotoxicity Associated with Dabrafenib Administration

M. Reid Groseclose^†^, Susan B. Laffan^‡^, Kendall S. Frazier^‡^, Angela Hughes-Earle^‡^, Stephen Castellino^†^

^†^ Drug Metabolism and Pharmacokinetics, GlaxoSmithKline, Research Triangle Park, NC 27709

^‡^Safety Assessment, GlaxoSmithKline, King of Prussia, PA 19406

**Supplementary Material**

**Formation of (M+2H+H)^+^ Ion for DAB and its Metabolites in MALDI MS**

When analyzed by MALDI MS, an ion corresponding to the [M+2H+H]^+^ is detected in addition to the [M+H]^+^ ion for DAB and its metabolites. For example, **Figure S1a** displays a spectrum acquired by MALDI MS for a standard solution containing DAB, DDAB and HDAB, acquired in positive ion mode using DHB matrix with the respective [M+H]^+^ and [M+2H+H]^+^ labeled for each species. As clearly shown in this figure, this [M+2H+H]^+^ species is detected with close to 2-fold higher intensity than the [M+H]^+^ peak for each of the metabolites. Interestingly, the abundance of this ion appears to be matrix and polarity dependent. It is observed with the highest intensity using 2,5-DHB (~2x [M+H]^+^), to a lesser extent with CHCA (~0.5x [M+H]^+^) and is not observed at all with 9-aminoacridine (9-AA) in negative ion mode.

Based on the fragmentation pattern generated by MS/MS on this species it was determined that it is likely generated by the opening of the pyrimidinamine ring (**Figure S1b**). For example, **Figure S2a** shows the respective fragmentation spectrum generated for DDAB [M+2H+H]^+^ (top) and [M+H]^+^ (bottom). The [M+2H+H]^+^ ion generates a unique fragment ion at *m/z* 466.0861, corresponding to a loss of formamidine from the ring-opened structure. This fragment is not observed in the MS/MS spectrum generated from the [M+H]^+^ ion. In contrast, the [M+H]^+^ ion generates several fragment ions that correspond to structures where the pyrimidinamine ring is intact.

The [M+2H+H]^+^ ion is not observed for DAB or its metabolites when analyzed using ESI. Given that the abundance of this ion appears to be matrix dependent, the mechanism driving the formation of this ion is likely due to analyte matrix interactions occurring during crystallization and/or desorption/ionization.


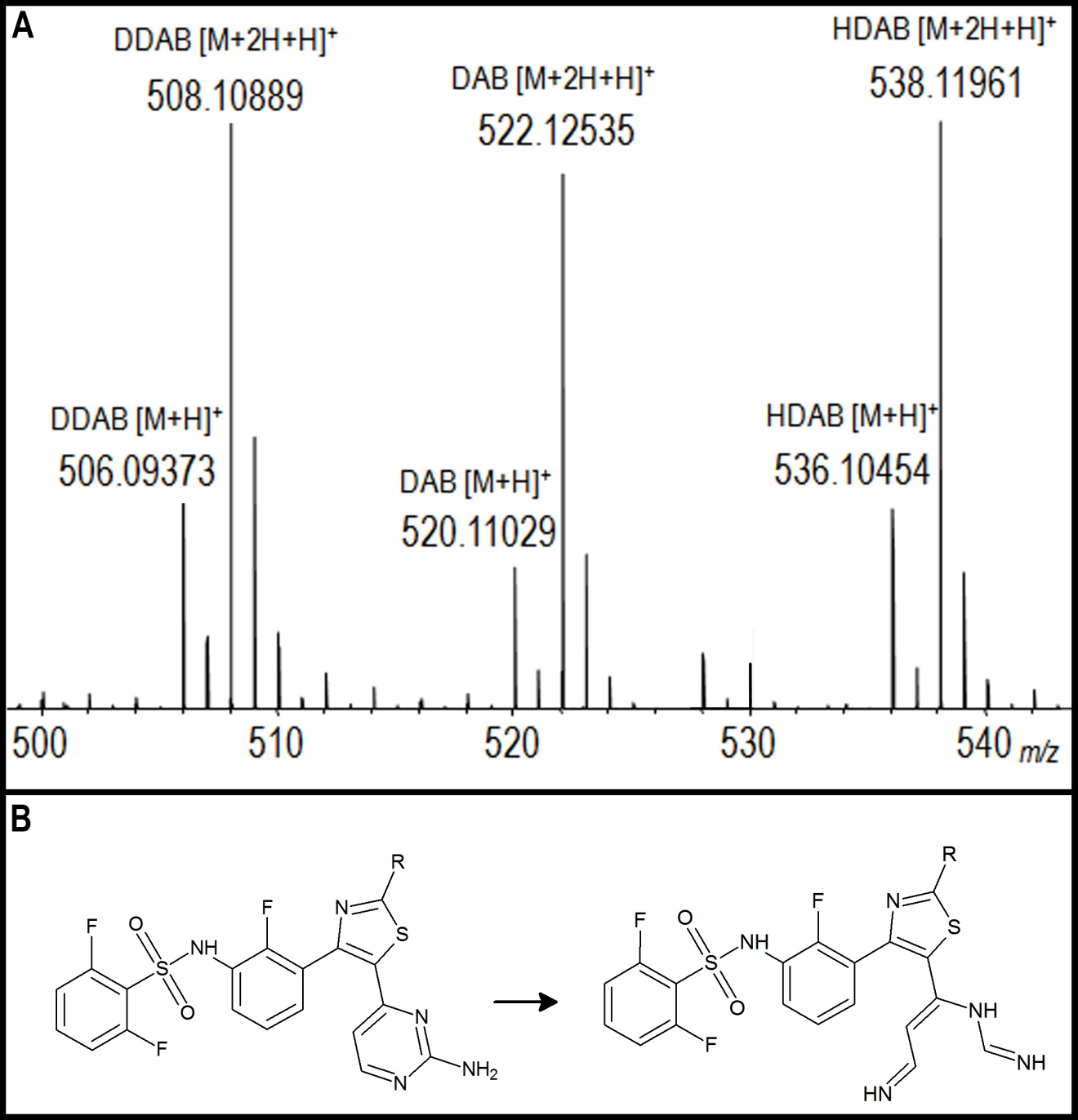


**Fig. S1 A)** MALDI mass spectrum acquired in CASI mode (m/z 460-620) for a standard mix containing DAB, DDAB and HDAB in positive ion mode using DHB **B)** Proposed pyrimidinamine ring opened structure for the [M+2H+H]^+^ species

**
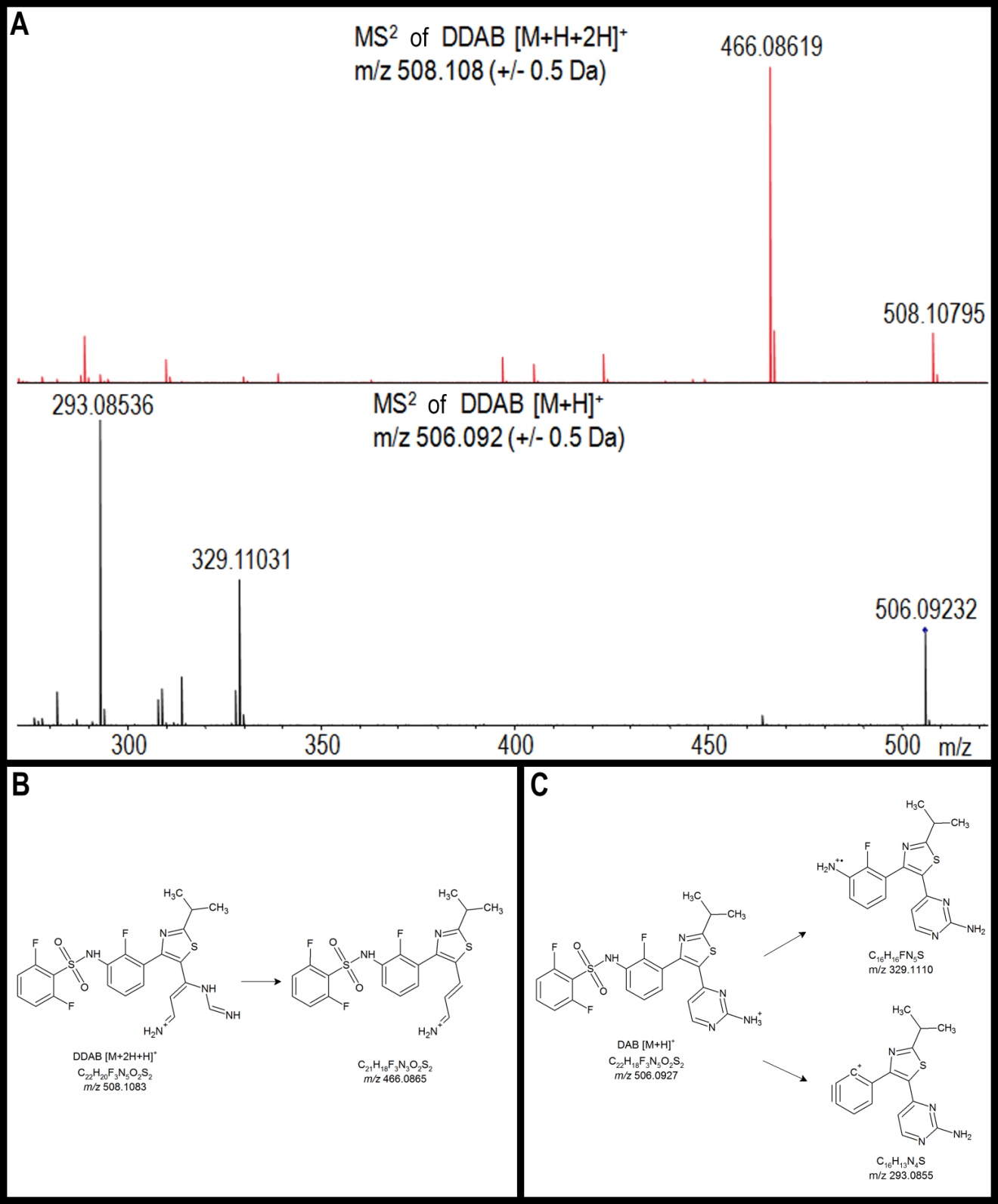
**

**Fig. S2 A)** MALDI MS/MS spectrum for DDAB [M+2H+H]^+^ (top) and [M+H]^+^ (bottom) acquired in positive ion mode using DHB **B)** Proposed fragment ion structures for the most abundant peaks in each spectrum

**Conversion of CDAB to DDAB**

When analyzed by MALDI MS, carboxy-dabrafenib (CDAB) undergoes extensive conversion to desmethyl-dabrafenib (DDAB) through decarboxylation. The percentage conversion of CDAB to DDAB (referred to CDAB* in text) was shown to be approximately 90% using a tissue mimetic model and directly comparing intensities for the respective ions (**Figure S3**). Notably, this percent conversion was observed to be consistent across a broad range of CDAB concentrations (1000-50,000 ng/g). The tissue mimetic model was generated using a previously described method[1], where spiked tissue homogenates are frozen into a polymer mold, cryosectioned and analyzed by MALDI IMS.

This conversion is specific to MALDI as it is not observed with ESI; however, it is unknown whether it occurs during sample preparation (e.g. during matrix application/crystallization) or during the desorption/ionization process (e.g. photochemically). Bershas *et al*.[2] reported that CDAB undergoes conversion to DDAB following *in vitro* incubation for 1 hr in aqueous buffer under acidic conditions (pH 3-4). The proposed mechanism is based on an acid catalyzed decarboxylation involving the aryl nitrogen of the thiazole ring as a Lewis base. The relevance of this mechanism to the decarboxylation observed by MALDI MS has not been investigated.

In the PND 7-13 kidney, LC-MS quantification showed that CDAB was the primary species with the highest concentration while the concentration of DDAB was negligible in comparison. Given that the conversion of CDAB to DDAB was shown to be consistent across a large range of concentration, the signal for DDAB was therefore used as a surrogate for CDAB and referred to CDAB* throughout the text.


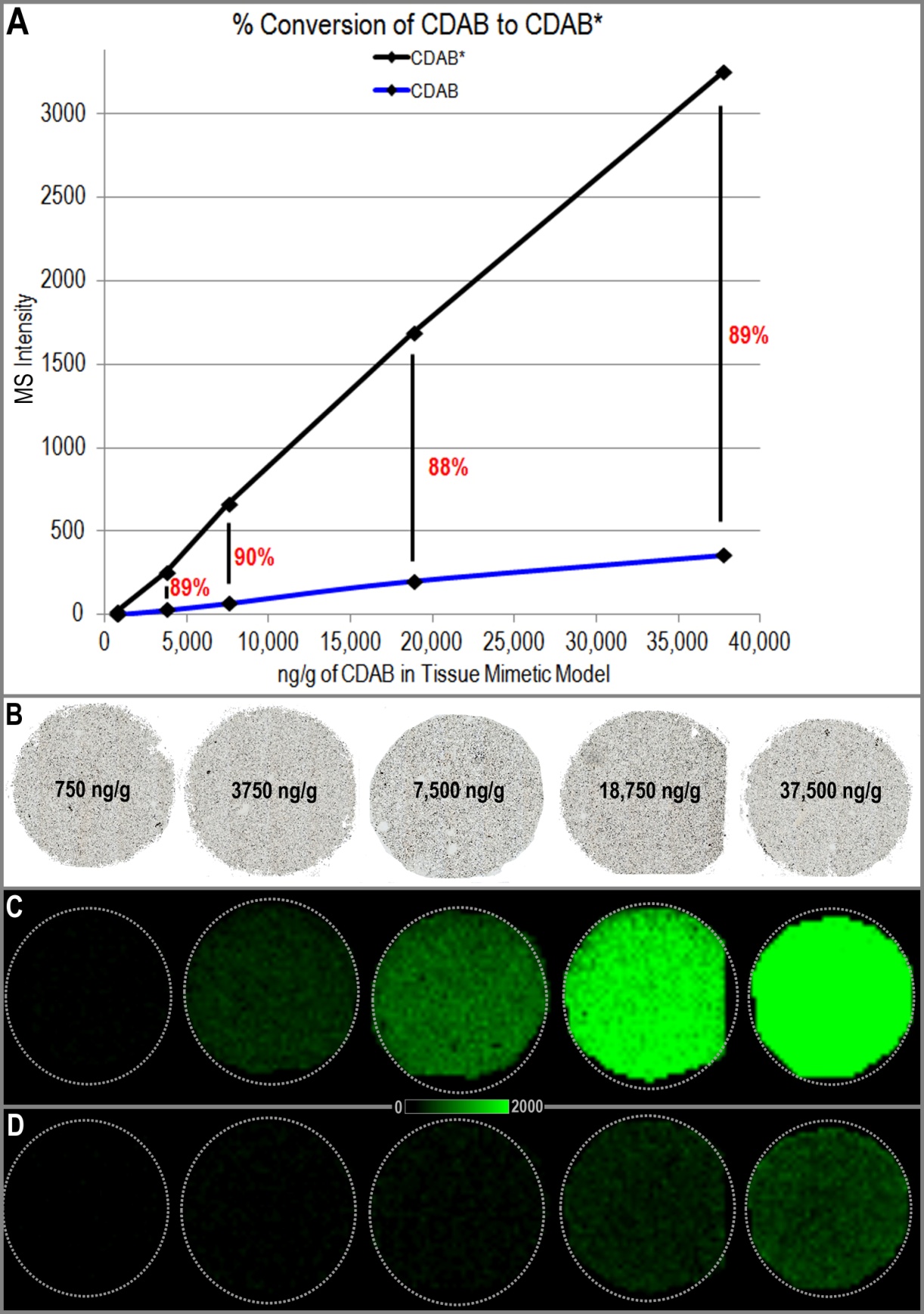


**Fig. S3 A)** Calibration plots for decarboxylated CDAB (CDAB*) and CDAB generated from a tissue mimetic model by MALDI IMS in CASI mode (m/z 460-620). Plotted intensity values for each concentration are generated by averaging all spectra within each core. **B)** Pre-analysis optical scan of tissue mimetic model cores with spiked concentration of CDAB labeled for each. Ion images for **C)** CDAB* and **D)** CDAB plotted at the same absolute intensity threshold

**LC-MS/MS of Kidney Tissue Homogenate Extracts**

One half of a frozen kidney tissue sectioned at the midline was collected from each animal into separate vials containing ceramic beads and 1 mL of methanol/water (3:1 v/v) was added to each. The tissues were homogenized/extracted three times using a FastPrep-24 (MP Biomedicals, Santa Ana, CA) for 3 x 60 sec. cycles at 6.5 m/sec, vortex mixed for 30 s and then centrifuged for 2 min. at 10,000 g. Supernatants from each extraction were combined and then dried down (Genevac Evaporator, Stone Ride, NY). The residues were reconstituted by sonication and vortex mixing following the addition of 500 μL of H_2_O/DMSO (9:1 v/v).

The standards for the LC-MS experiment were generated by spiking aliquots of the control kidney extracts with a range of standard concentrations. Two separate sets of standards were prepared: Set 1) three extracts each containing DAB, CDAB, HDAB and DDAB at 250 ng/mL, 125 ng/mL, and 62.5 ng/mL and Set 2) two extracts containing CDAB at 3.33 μg/mL and 1.66 μg/mL. Standard Set 1 was used for quantification of DAB, HDAB and DDAB and Set 2 was used for quantification of CDAB.

Aliquots of each extract were analyzed by LC-MS using ESI on an Orbitrap XL mass spectrometer (Thermo Scientific, Waltham, MA) in positive ion mode coupled with an 1260 Infinity LC (Agilent, Santa Clara, CA). Separations were performed using a linear gradient (solvent A – H_2_O w/ 10 mM ammonium acetate adjusted to pH 5.5 w/ acetic acid); solvent B - acetonitrile) on a Phenomenex Synergi C18 column (4 µm, 4.6 mm X 250 mm) at a flow rate of 1 mL/min. The peak areas from the extracted ion chromatograms (XIC) for the standards were used to generate individual calibration curves for DAB, CDAB, HDAB and DDAB. The peak area for the DAB-related material in the XIC of the tissue samples was compared to the appropriate calibration curve to estimate the quantities of each DAB-related component in the kidney homogenate from each animal.

The estimated limit of detection for DAB, HDAB, and DDAB was 50 pg on column and the limit of quantification was 250 pg. For CDAB, the estimated limit of detection was 500 pg on column and the limit of quantification was 2.5 ng. Based on the average tissue homogenate extract concentration (~ 0.5 g tissue / mL) and injection volume (0.050 mL), the tissue level LOD and LOQ for CDAB are estimated to be 20 ng/g and 100 ng/g, respectively. For DAB, HDAB, and DDAB the tissue level LOD and LOQ are estimated to be 2 ng/g and 10 ng/g, respectively.

**
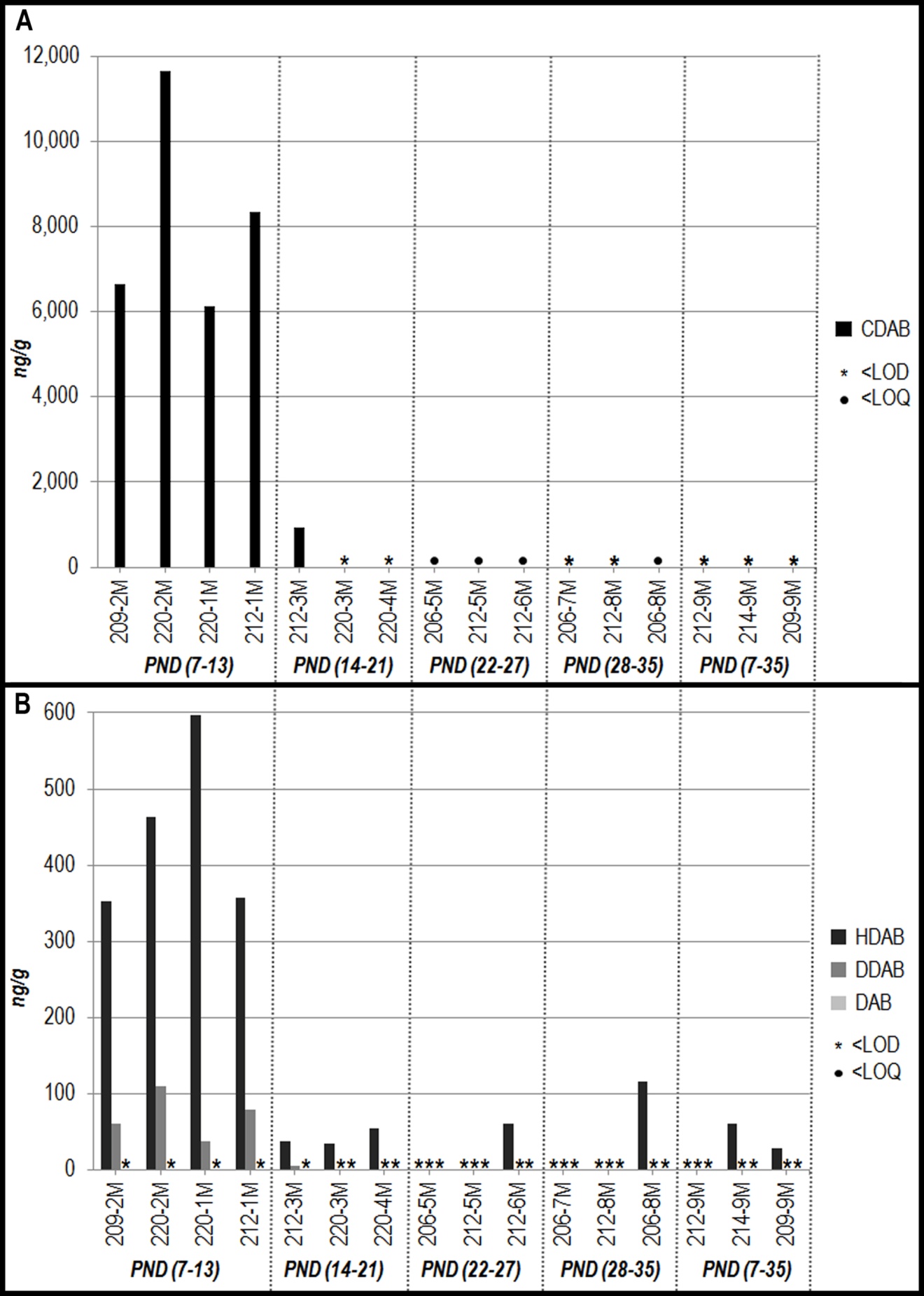
**

**Figure S4 a)** Concentrations of CDAB and **b)** HDAB, DDAB and DAB in Juvenile Rat Kidney Tissue Homogenate Extracts Detected by LC-MS

Supplementary Information References

1. Groseclose, M.R. and S. Castellino, *A mimetic tissue model for the quantification of drug distributions by MALDI imaging mass spectrometry.* Anal Chem, 2013. **85**(21): p. 10099-106.

2. Bershas, D.A., et al., *Metabolism and disposition of oral dabrafenib in cancer patients: proposed participation of aryl nitrogen in carbon-carbon bond cleavage via decarboxylation following enzymatic oxidation.* Drug Metab Dispos, 2013. **41**(12): p. 2215-24.
